# Influence of teacher autonomy support in feedback on high school students’ feedback literacy: the multiple mediating effects of basic psychological needs and intrinsic motivation

**DOI:** 10.3389/fpsyg.2024.1411082

**Published:** 2024-08-13

**Authors:** Shang Zhang, Jie Xu, Hao Chen, Lan Jiang, Xinfa Yi

**Affiliations:** ^1^Department of Education, Shaanxi Normal University, Xi’an, China; ^2^Key Laboratory of Modern Teaching Technology (Ministry of Education), Shaanxi Normal University, Xian, China

**Keywords:** Chinese high school students, teacher autonomy support, basic psychological needs, intrinsic motivation, feedback literacy

## Abstract

This research presents and confirms an intermediary model, deeply anchored in self-determination theory, to dissect the influence of Chinese high school students’ core psychological needs and intrinsic drive on the nexus between educators’ autonomous backing and students’ proficiency in feedback literacy, highlighting the mediating roles of these elements. A survey of 704 Chinese senior high school students, including 319 males and 385 females, employed the Feedback Literacy Scale, Basic Psychological Needs Scale, Intrinsic Motivation Scale, and Perceived Teacher Autonomy Support Scale. The study’s discoveries illuminate that educators’ autonomous support not only directly amplifies students’ feedback literacy but also has an indirect impact through the intermediation of basic psychological needs and intrinsic motivation, along with their interconnected dynamics. This inquiry not only deepens our grasp of the mechanisms interlinking teacher support with feedback literacy but also critically evaluates the findings to proffer targeted recommendations, thereby enhancing our comprehension of the underlying processes and guiding educational practices and student development.

## Introduction

Feedback literacy is considered to be the pivotal factor influencing the disparity in students’ academic performance and innovative accomplishments (Bolden et al., 2020; Chen et al., 2024). Having a high level of feedback literacy not only ensures excellent academic performance (Hattie and Timperley, 2007), but also enhances students’ foundational literacy for future work and lifelong learning (Noble et al., 2020). Therefore, the topic of how to provide feedback to students and how students should assimilate feedback has thus become a focal point for numerous researchers (Molloy and Boud, 2012; Chong, 2021).

In this field research pioneers have conducted a series of empirical studies to explore how different teaching strategies to support feedback can improve students’ feedback literacy. For example, teacher feedback intervenes with peer feedback (Han and Xu, 2020), feedback input toolkit as an intermediary resource (Winstone et al., 2019), and conversational feed forward evaluation (Hill and West, 2019). Compared with the traditional teacher-student feedback and peer feedback, these studies emphasize the role of teachers’ autonomous support in the feedback process. However, further investigation is warranted to explore the impact of teacher autonomy support on students’ feedback literacy in a more comprehensive manner. Specifically, it is crucial to determine the strength of this relationship and identify potential factors that may influence it. These aspects necessitate additional analysis and discussion.

In addition, since most of the existing studies focus on the higher education stage, the attention on the feedback literacy of students in the basic education stage is insufficient. Therefore, this study aims to explore and verify the influence of teacher autonomy support in feedback on senior high school students’ feedback literacy and the role of its mediating factors. Through this research, we hope to fill the gap in the research of students’ feedback literacy in the basic education stage, and provide empirical evidence for the educational practice of teachers’ autonomous support and students’ feedback literacy.

### Student feedback literacy

In recent years, the academic community has generally accepted the definition of student feedback literacy proposed by Carless and Boud (2018). This definition states that in order to understand feedback and use it to improve work or learning strategies, students need to possess qualities of understanding, competence and temperament. Students’ feedback literacy includes four elements: Sensing feedback, making judgments, managing emotions and taking actions. Perceiving feedback means that students need to recognize the meaning and value of feedback and appreciate the positive role in the feedback process. Making judgments requires students to learn to make reasonable judgments about their own work and that of others. Managing emotions means that students need to effectively manage emotional responses to feedback. These three elements focus on taking action on this step in order to contribute to improving future work (Carless and Boud, 2018; Chong, 2021).

Students’ feedback literacy is formed and developed in feedback practice, which also restricts the current level of feedback practice (Carless and Boud, 2018; Chong, 2021). In other words, feedback literacy and feedback practice form a relatively closed and stable system, which may hinder the further development of feedback literacy. Therefore, in order to continuously improve students’ feedback literacy, it is necessary to inject new energy into the feedback literacy—feedback practice circular system. To date, Chong’s (2021) eco-feedback literacy framework is one of the first specialized studies to explore this energy. Chong (2021) points out that factors influencing feedback literacy include external-environmental factors (such as feedback texts, interpersonal relationships, teaching environment, and sociocultural) as well as internal-personal factors (such as beliefs, goals, experiences, and abilities).

However, although Chong’s (2021) study provided many hypotheses for exploring the influencing factors of feedback literacy, it lacked empirical research support and did not explore the relationship between various factors. Therefore, this study will further track how teacher teaching and student competence together influence student feedback literacy.

### Teacher autonomy support

Teacher autonomy support refers to the way that teachers respect students’ free choice and independent decision in teaching. This kind of support encourages students to think independently, solve problems, and provide necessary information and options, while understanding students’ inner feelings and avoiding pressure demands (Deci and Ryan, 2000; Mageau and Vallerand, 2003; Ryan and Deci, 2020). In teaching practice, teachers’ autonomous support can be embodied in three types: using non-controlling verbal information to communicate; acknowledge and understand students’ perspectives and emotions; identify and develop students’ intrinsic motivational resources, including psychological needs, interests, preferences, goals, and values (Reeve and Jang, 2006; Jang et al., 2010).

Compared with the control environment, when students perceive the teacher’s autonomous support, they will show more interest in learning, have more pleasure in school life, show higher learning effort and persistence, and therefore be more engaged in learning (Reeve et al., 2004). Teachers’ autonomous support can not only directly promote students’ healthy behavior and literacy accumulation, but also play a role in satisfying students’ basic psychological needs and stimulating internal motivation (Deci and Ryan, 2000; Ryan and Deci, 2020). Therefore, teacher autonomy support is considered to be a learner-specific environmental resource with the strongest predictive power of students’ motivational styles and educational achievement (Ma, 2021).

### Teacher autonomy support and student feedback literacy in feedback

The purpose of teacher autonomous support in feedback is to give students more respect, choice, incentive and support, so as to promote students’ active participation in the feedback process and effectively absorb feedback. Nowadays, more and more researchers have gradually realized that the old paradigm of teacher-centered process-oriented feedback has multiple problems and drawbacks, and advocated a new paradigm of student-centered process-oriented feedback (Ajjawi and Regehr, 2019; Chong, 2021). At the heart of the new paradigm lies the teacher’s respect for students’ subjectivity and the autonomy to mobilize students to ensure that students actively participate in feedback and get the maximum benefit from it (Nieminen and Carless, 2023).

At the same time, studies in many fields have shown that independent choice is the fundamental reason for the development process of individuals from not active to active participation (Mullan and Markland, 1997; Wilson and Rodgers, 2002). In the new paradigm, the feedback process extends beyond passive reception to active construction. Students, peers, and teachers all play multiple roles: they are senders, receivers, and negotiators of information, as well as constructors and creators of their own feedback meanings and beliefs. As the beneficiaries of feedback, students actively participate in feedback through interaction with teachers, peers and technical support, as well as self-reflection, and fully enjoy the benefits of feedback (Ajjawi and Regehr, 2019; Winstone et al., 2019; Chong, 2021; Nieminen and Carless, 2023). Based on the above views, we proposed hypothesis:

H1: Teacher autonomy support in feedback has a positive predictive effect on high school students’ feedback literacy.

### The role of basic psychological needs and internal motivation in the relationship between teachers’ autonomous support and students’ feedback literacy

According to self-determination theory, people have three basic psychological needs: autonomy, competence and relevance, which exist in any social situation. When these psychological needs are met, social situations promote the development of internal motivations and facilitate the internalization of external motivations. This kind of satisfaction can enhance the individual’s sense of happiness and achievement when participating in activities, and then promote the individual to develop in a positive and healthy direction. On the contrary, if these psychological needs are not met, the individual may develop in a negative direction or develop functional impairment (Deci and Ryan, 2000; Jang et al., 2012).

The research shows that teachers’ autonomous support in the feedback process has a direct impact on students’ feedback literacy, and there may also be a potential indirect effect. It is interesting to explore the significance of this indirect effect. To this end, this study aims to investigate the influence of teacher autonomy support in feedback on students’ feedback literacy, and further reveal the multiple mediating mechanisms of this association.

Basic psychological needs include the need for autonomy, mastery and relevance. The need for autonomy refers to the ability of an individual to choose to engage in behavioral activities independently. The need for competence refers to the belief that an individual can achieve the expected result through efforts. The need for relevance refers to the need of individuals to establish close emotional bonds and attachments with others, reflecting the expectation of establishing connections with important people in their lives (Levesque et al., 2004).

Research has confirmed that teacher autonomy support can not only meet students’ autonomy needs, but also meet students’ competency and relevance needs to a certain extent (Chicote-López et al., 2018). Krijgsman et al. (2019) found that in terms of goal clarification and process feedback, teachers are positively correlated with students’ experience of satisfying their basic psychological needs, while negatively correlated with students’ frustration with their basic psychological needs. Mouratidis et al. (2010) both showed that providing corrective feedback in a way that supports students’ autonomy can better meet students’ basic psychological needs and enhance students’ subjective vitality. In addition, the framework of teacher feedback literacy further elaborates how teacher’ autonomous support in feedback meets students’ feedback needs and promotes the development of students’ feedback literacy. Carless and Winstone’s (2020) framework on teacher feedback literacy emphasizes the role of teacher autonomy support in the feedback process. This support involves fulfilling students’ needs for autonomy, relatedness, and competence through design, relationship, and practice.

Therefore, teachers’ autonomous support in feedback can provide students with a variety of time, space and sources, and provide continuous and coherent feedback support. Based on the above discussion, we propose hypothesis:

H2: Basic psychological needs play a mediating role in the influence of teachers’ autonomous support in feedback on senior high school students’ feedback literacy.

In addition to basic psychological needs, intrinsic motivation may play an important role in this process. Ryan and Deci (2020) defined intrinsic motivation as motivation derived from an individual’s intrinsic interest and enjoyment of the work itself, rather than external rewards or accolades (Amabile, 1993). They explain that intrinsic motivation is driven by three key needs in individual behavior: the need for value (such as self-improvement or self-worth), the need for ability (referring to the desire for task success or mastery), and the need for pleasure (relating to the enjoyment or happiness experienced during an activity). In addition, other research supports and complements these ideas (Deci et al., 1994; Cerasoli et al., 2014).

This intrinsic motivation influences task performance in a variety of ways, including the individual’s high concentration on a task, effort to achieve success, and persistence during task execution (Cerasoli et al., 2014). Research has shown that individuals with higher intrinsic motivation are stronger in terms of focus, persistence, and willingness to work hard (Grant and Berry, 2011), which makes them more proactive and active in the feedback process, and promotes their absorption of feedback and the formation of feedback literacy.

Research also shows that teacher autonomy support in feedback can effectively enhance students’ intrinsic motivation. Research by Mabbe et al. (2018) found that providing positive normative feedback in a way that supports autonomy produces the most positive motivational outcomes for students’ intrinsic motivation. Similarly, Hagger et al. (2015) found that both autonomous causal orientation and positive competence enhancement feedback can enhance intrinsic motivation. Therefore, this study proposed hypothesis:

H3: Intrinsic motivation plays an intermediary role between teacher autonomy support in feedback and high school students’ feedback literacy.

In addition, self-determination theory points out that the satisfaction of basic psychological needs can positively predict intrinsic motivation, and they can also work together to promote students’ healthy behaviors and literacy accumulation (Orsini et al., 2018). Orsini et al.’s research found that basic psychological needs play a mediating role between the atmosphere supporting autonomous learning, the quantity and quality of feedback received, and students’ autonomous motivation. Zhang and Qian (2022) found the chain-mediated effect of basic psychological needs and autonomous motivation between teachers’ sense of support for obese students and their involvement in sports learning. Therefore, this study proposes hypothesis:

H4: Basic psychological needs and intrinsic motivation play a chain mediating role between teacher autonomy support in feedback and high school students’ feedback literacy.

## Method

### Participants

For this study, a stratified sampling method was employed to select a diverse and representative group of high school students from A High School in a city of Shanxi Province, China. The school’s enrollment policy caters to the entire city, ensuring that the sample sources are geographically dispersed and reflective of the broader population. The inclusion criteria for our sample were set to encompass students who were currently enrolled at the school, actively participating in their academic curriculum, and willing to provide informed consent for their participation in the study. Exclusion criteria were established to exclude students with significant cognitive impairments or those unable to complete the survey due to language barriers or other disabilities. A total of 720 high school students were invited to participate in the survey. After rigorously applying the exclusion criteria and removing 16 invalid questionnaires reported by the participants, 704 valid questionnaires were obtained (*M* = 17.38, *SD* = 1.09), representing an effective response rate of 97.78%. The demographic breakdown of the sample included male students (*n* = 319, *M* = 17.45, *SD* = 1.10) and female students (*n* = 385, *M* = 17.32, *SD* = 1.08), with representation across different academic years: Senior one (*n* = 306, *M* = 16.47, *SD* = 0.61), Senior two (*n* = 202, *M* = 17.60, *SD* = 0.66), and Senior three (*n* = 196, *M* = 18.58, *SD* = 0.67).

## Materials

### Feedback literacy scale (FLS)

The Feedback Literacy Scale developed by Zhan (2022) was adopted. Chinese scholar Chen et al. (2024) directly applied the Chinese version of the questionnaire to measure the feedback literacy of Chinese high school students, and verified that it has good reliability. Based on this, this study directly used the Chinese version of the questionnaire for measurement. The scale has 6 observational dimensions, with four questions in each dimension: eliciting, processing, enacting, appreciation of feedback, readiness to engage and commitment to change. Likert-6 points were used to score, 1–6 representing “strongly disagree—strongly agree,” and the arithmetic average of all questions was calculated. The higher the score, the better the feedback literacy of students (Zhan, 2022). In this study, the questionnaire had a *Cronbach’s Alpha* of 0.959 and a *KMO* of 0.963.

### Perceived teacher autonomy support scale (PTA)

The present study employs the Perceived Teacher Autonomy Support Questionnaire, initially compiled by Williams and Deci (1996) and later revised by Chinese scholar Chen (2008). This scale is unidimensional and comprises 15 items. Utilizing a Likert scale ranging from 1 to 7, where 1 signifies “strongly disagree” and 7 signifies “strongly agree,” the arithmetic mean of all items is computed. A higher score indicates a stronger perception of teacher support for autonomy among students. Based on an analysis of extreme value comparisons and the “Modification Indices” suggested by AMOS24.0, one item—“I do not quite like the way my teacher speaks to me (*R*)”—was deleted. Following the removal of this item, the questionnaire’s *Cronbach’s Alpha* value and *KMO* value for this scale were found to be 0.961 and 0.965, respectively.

### Basic psychological needs scale (BPNS)

The Basic Psychological Needs Scales, originally developed by Gagné (2003) and subsequently revised by Chinese scholars Liu et al. (2013), were directly utilized in this study. The scale encompasses three dimensions with a total of 19 items: Autonomy needs, Competency requirements, Ownership needs.

The scale employs a 6-point Likert scale for scoring, where 1 represents “completely disagree” and 6 represents “completely agree.” The arithmetic mean of all items is calculated, with higher scores indicating a greater degree of satisfaction. In this study, the *Cronbach’s Alpha* value and *KMO* measure of sampling adequacy for this scale were found to be 0.843 and 0.879, respectively.

### Intrinsic motivation scale (IMS)

The Intrinsic Motivation Scale, developed by Isen and Reeve (2005), was utilized in this study. The questionnaire has been localized by Chinese scholars Gong et al. (2017) and has demonstrated good reliability and validity. The scale is unidimensional and consists of 7 items. It uses a 6-point Likert scale, where 1 represents “completely disagree” and 6 represents “completely agree.” The arithmetic average of all items is calculated, and the higher the score, the stronger the learning motivation. In this study, the *Cronbach’s Alpha* value and *KMO* measure of sampling adequacy for this scale were found to be 0.963 and 0.938, respectively.

### Procedure

The research was fully executed in May 2023, with doctoral candidates from the School Curriculum and Instruction program, who were meticulously trained, taking on the role of lead evaluators. Before commencing the assessments, we secured informed consent from both the parents and the students involved, underscoring the importance of maintaining confidentiality. The scales, including the Perceived Teacher Autonomy Support Scale, Basic Psychological Needs Scale, Intrinsic Motivation Scale, and Feedback Literacy Scale, were administered in an orderly fashion, instructing participants to answer based on their immediate reactions and true experiences. The duration of the assessment was approximately 30 min, and it was carried out in a collective format within the classroom setting.

### Data analysis

Based on the need to verify the research hypothesis, this study designed the following data processing and analysis links. Firstly, invalid questionnaires were eliminated according to the methods of students’ self-report and boxplot test. Secondly, *SPSS22.0* software is used for common method deviation test, descriptive statistics, correlation analysis and regression analysis. Finally, *AMOS24.0* software was used to construct a structural equation model, and the relationship between teachers’ autonomous support, basic psychological needs, intrinsic motivation and feedback literacy in the feedback of sample groups was gradually tested.

## Result

### Test of common method bias

Harman’s single-factor test (Podsakoff and Organ, 1986) was used to conduct common method bias. Perform unrotated principal component analysis. Among them, there are 9 factors with eigenvalues greater than 1, and the variance interpretation variance of the first factor is 35.11%, which is lower than 40%, indicating that the common method deviation of this study is not serious and will not affect the research conclusion.

### Descriptive statistics and correlation analysis

This study uses Pearson correlation analysis to explore the relationship between teachers’ autonomous support, basic psychological needs, intrinsic motivation and high school students’ feedback literacy. The results show (refer to Table 1) that there is a significant correlation between each variable and its internal factors.

**Table 1:** Descriptive statistics and correlation analysis of each variable.

	*M*	*SD*	FL	El	Pr	En	AOF	RTO	CTC	IM	BPN	ON	CR	AN	TAS
FL	4.36	0.70	1												
El	4.40	0.83	0.838^***^	1											
Pr	4.39	0.80	0.872^***^	0.708^***^	1										
En	4.26	0.85	0.872^***^	0.708^***^	0.746^***^	1									
AOF	4.45	0.80	0.885^***^	0.667^***^	0.747^***^	0.749^***^	1								
RTO	4.26	0.90	0.808^***^	0.574^***^	0.623^***^	0.573^***^	0.667^***^	1							
CTC	4.43	0.76	0.852^***^	0.639^***^	0.657^***^	0.698^***^	0.719^***^	0.674^***^	1						
IM	4.76	1.22	0.635^***^	0.587^***^	0.564^***^	0.549^***^	0.557^***^	0.464^***^	0.541^***^	1					
BPN	4.48	0.70	0.490^***^	0.442^***^	0.432^***^	0.444^***^	0.434^***^	0.370^***^	0.389^***^	0.375^***^	1				
ON	4.88	0.89	0.387^***^	0.367^***^	0.355^***^	0.314^***^	0.365^***^	0.285^***^	0.298^***^	0.299^***^	0.850^***^	1			
CR	4.31	0.81	0.448^***^	0.408^***^	0.394^***^	0.437^***^	0.369^***^	0.329^***^	0.359^***^	0.355^***^	0.852^***^	0.552^***^	1		
AN	4.25	0.78	0.427^***^	0.360^***^	0.362^***^	0.396^***^	0.381^***^	0.341^***^	0.346^***^	0.312^***^	0.866^***^	0.599^***^	0.648^***^	1	
TAS	4.73	1.05	0.620^***^	0.586^***^	0.529^***^	0.508^***^	0.510^***^	0.495^***^	0.554^***^	0.537^***^	0.483^***^	0.416^***^	0.420^***^	0.403^***^	1

^***^Indicates significance level *p*<0.001.FL indicates Feedback literacy, El indicates Eliciting, Pr indicates processing, En indicates enacting, AOF indicates appreciation of feedback, RTE indicates Readiness to engage, CTC indicates Commitment to change, IMS indicates Intrinsic motivation, BPN indicates Basic Psychological Needs, ON indicates Ownership Needs, CR indicates Competency Requirements, AN indicates Autonomy Needs, TAS indicates Teacher Autonomy Support.

### Mediation analysis

In order to preliminarily verify hypothesis H1, with teacher autonomy support in feedback as the independent variable and feedback literacy of senior high school students as the dependent variable, SPSS 22.0 was used for regression analysis. The results showed that teacher autonomy support in feedback could significantly and positively correlate with their feedback literacy (*F* = 439.347, *R*^2^ = 0.385, *β* = 0.620, *t* = 20.961, *p* < 0.001), that is, the higher the level of teacher autonomy support in feedback, the higher the level of feedback literacy of senior high school students.

To further validate research hypotheses H1, H2, H3, and H4, we utilized *AOMS 24.0* software to construct models using the stepwise method. We adopted the stepwise methodology (Baron and Kenny, 1986) to establish model M1 without mediation and its modified version M1a, model M2 and M3 with single mediation, model M4 with a chain mediation, and model M5 with multiple mediation (Table 2). The stepwise approach allows us to clearly observe the process of sequentially adding mediating variables, which helps to better understand the relationships between variables. Additionally, the model was modified by adding a covariant relationship of error variables within the same factor, based on the information provided by “Modification Indices.” Moreover, we used Bootstrap self-sampling with 2000 replications, and set the Confidence Interval (*CI*) to 95%. The *CI* [Lower, Upper] excluding 0 was used as an index to test the effect size.

**Table 2:** Significance test and effect value of each model fitness indices and mediation effect based on stepwise test method.

Model	*χ^2^/df*	*CFI*	*TLI*	*RMSEA*	*SRMR*	Pathway	B	95%CI	*B*%
								[Lower, Upper]	
M1	5.840	0.932	0.924	0.083	0.039	TSA → FL	0.639	[0.323, 0.438]	100.00
M1a	4.340	0.955	0.947	0.069	0.037	TSA → FL	0.634	[0.314, 0.430]	100.00
M2	3.681	0.955	0.948	0.062	0.036	TSA → FL	0.274	[0.213, 0.339]	74.05
						TSA → BPN → FL	0.096	[0.070, 0.130]	25.95
M3	3.566	0.955	0.950	0.060	0.034	TSA → FL	0.229	[0.181, 0.280]	61.39
						TSA → IM → FL	0.144	[0.110, 0.183]	38.61
M4	2.929	0.923	0.916	0.071	0.037	TSA → FL	0.238	[0.177, 0.309]	79.60
						TSA → BPN → IM → FL	0.061	[0.035, 0.095]	20.40
M5	2.758	0.930	0.923	0.068	0.041	TSA → FL	0.175	[0.107, 0.257]	49.44
						TSA → BPN → FL	0.070	[0.031, 0.114]	19.77
						TSA → IM → FL	0.092	[0.056, 0.134]	25.99
						TSA → BPN → IM → FL	0.017	[0.003, 0.036]	4.80

TSA indicates Teacher autonomy support, FL indicates Feedback literacy, BPN indicates Basic psychological needs, IM indicates Intrinsic motivation.

**Figure 1:**
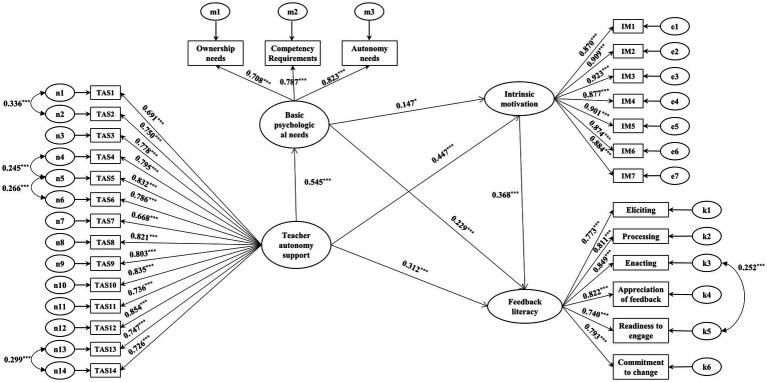
Multi-mediating model of teacher autonomy support in feedback of basic psychological needs and intrinsic motivation to students’ feedback literacy.

The results indicate that the fitting indices for each model are good (Table 2). In model M5 (Figure 1), teacher autonomy support in feedback is positively correlated with the basic psychological needs, intrinsic motivation, and feedback literacy of high school students (*β* = 0.545, *p* < 0.001; *β* = 0.447, *p* < 0.001; *β* = 0.312, *p* < 0.001). Basic psychological needs are positively correlated with intrinsic motivation and feedback literacy (*β* = 0.147, *p* < 0.05; *β* = 0.229, *p* < 0.001), and intrinsic motivation is positively correlated with feedback literacy (*β* = 0.368, *p* < 0.001). The significance of these path coefficients only indicates the existence of a relationship. To confirm the mediating effects, further mediation analysis is required.

The Bootstrap test (Table 2) revealed that teacher autonomy support in feedback had a significant direct effect on high school students’ feedback literacy (*B* = 0.175, *CI* [0.107, 0.257]), accounting for 49.44% of the effect size, which supports hypothesis H1. The mediating effect of teacher autonomy support on students’ feedback literacy through basic psychological needs was significant (*B* = 0.070, *CI* [0.031, 0.114]), accounting for 19.77% of the effect size, supporting hypothesis H2. The mediating effect of intrinsic motivation on the relationship between teacher autonomy support and student feedback literacy was also significant (*B* = 0.092, *CI* [0.056, 0.134]), with an effect size of 25.99%, supporting hypothesis H3. Additionally, there were significant chain mediation effects of basic psychological needs and intrinsic motivation on the relationship between teacher autonomy support and students’ feedback literacy (*B* = 0.017, *CI* [0.003, 0.036]), accounting for 4.80% of the effect size, which also supports hypothesis H4. It is evident that both basic psychological needs and intrinsic motivation play multiple mediating roles in the influence of teacher autonomy support on students’ feedback literacy.

## Discussion

### Teacher autonomy support and high school students’ feedback literacy in feedback

The research findings align with Hypothesis 1 and provide additional support for the conceptualization of a student-centered, process-oriented feedback paradigm (Ajjawi and Regehr, 2019; Chong, 2021; Nieminen and Carless, 2023). These results suggest that teacher autonomy support within the feedback process is associated with enhanced feedback literacy among senior high school students.

This influence can be explained from the following three aspects: First, teacher autonomy support and respect for students’ subjectivity in the feedback process motivate students to play multiple roles in the feedback process, including interpersonal roles (sender, receiver and negotiator of feedback information) and cognitive roles (constructor, critic and re-creator of feedback information). In this way, students can participate in feedback in multiple capacities, perceive feedback from different perspectives, and internalize feedback. Secondly, in the feedback, teacher autonomy support attaches importance to students’ independent choice, avoids external control and pressure, and respects students’ inner feelings. In doing so, students are encouraged to engage in deeper information exchange, problem solving, and meaning reconstruction, allowing them to examine, evaluate, and utilize feedback on multiple levels. Finally, teacher autonomy support in feedback provides cognitive scaffolding and emotion regulation strategies for students, which helps students better internalize feedback, resolve conflicts, and manage emotions.

In light of the preceding discussion, the outcomes of this study underscore the significance of teacher autonomy support as a facilitator of feedback literacy growth among high school students. These findings hold practical implications for educational interventions and offer valuable insights for further investigation within the realm of educational psychology.

### The mediating role of basic psychological needs

The research findings support H2, suggesting that teacher autonomy support in feedback is associated with the satisfaction of students’ basic psychological needs, which in turn facilitates the development of their feedback literacy. This conclusion resonates with the findings of studies by Krijgsman et al. (2019) and Mouratidis et al. (2010). This study posits that beyond the direct influence of teacher autonomy support on students’ feedback literacy, the satisfaction of students’ needs for autonomy, competence, and relatedness serves as a mediating factor. Specifically, teacher autonomy support in feedback appears to meet students’ requirements for independent choice in feedback, active participation in feedback processes, independent reflection on feedback, and using feedback outcomes to serve their own needs. Furthermore, it addresses students’ needs for a variety of abilities, including cognitive scaffolding and emotional regulation strategies, as well as their needs for effective interpersonal interaction and conflict resolution. When these needs are fulfilled, students’ sense of ownership, agency, competence, community belonging, and interpersonal connection is enhanced (Ryan and Deci, 2020), making them more likely to engage proactively in feedback, absorb it effectively, and thereby enhance their feedback literacy.

### The mediating role of intrinsic motivation

The research findings support H3, demonstrating that teacher autonomy support in feedback can activate students’ intrinsic motivation, which in turn fosters the development of their feedback literacy. This aligns with the findings of Mabbe et al. (2018) and Hagger et al. (2015). This study also reveals that teacher autonomy support exerts its influence not only directly but also by mediating the enhancement of students’ perceived self-interest, task competence, and enjoyment (Deci et al., 1994). Specifically, self-selection empowers students to determine the focus and content of feedback based on their personal interests and areas for skill development, thereby enhancing their sense of self-benefit. Ability support assists students in overcoming feelings of helplessness, appreciating the cognitive scaffolding and emotional regulation strategies provided by teachers, and cultivating a sense of task competence. Moreover, by using non-controlling language and acknowledging negative emotions, teachers can attend more effectively to students’ affective experiences (Lan and Mastrotheodoros, 2022), thereby ensuring students’ sense of enjoyment and improving their emotional engagement with feedback, while mitigating potential cognitive-emotional conflicts. When these intrinsic motivations are engaged, students exhibit greater focus, increased effort, and sustained participation, leading to more effective absorption of feedback content and enhanced accumulation of feedback literacy.

### Chain mediation between basic psychological needs and internal motivation

The research findings support H4, showing that teacher autonomy support in feedback positively affects the satisfaction of students’ basic psychological needs, which in turn stimulates their intrinsic motivation and facilitates the development of feedback literacy. This concurs with the findings of Orsini et al. (2018) and Zhang and Qian (2022), who suggest that in the feedback process, teachers who provide an environment rich in autonomy support enable students to perceive this support. Such an environment not only enhances students’ sense of belonging but also makes them feel competent in engaging with feedback and experience a strong sense of autonomy. When students’ needs for autonomy, competence, and relatedness are met, they perceive feedback activities as self-determined, thereby enhancing their autonomous motivation to engage with feedback and promoting their ability to absorb and develop feedback literacy.

### Recommendations and limitations

This study faces the following major limitations: First, this study uses a self-rating scale to measure teacher autonomy support, and there may be differences between students’ perceived autonomy support and real teacher autonomy support. Future studies may consider combining objective assessment methods, such as observation of teaching scenarios and teacher interviews, to more comprehensively assess the extent of teachers’ autonomous support.

Secondly, the current study focuses on students from a high school in central China, which limits the sample size and scope of the research, potentially restricting the generalizability of the findings. To enhance the representativeness and external validity of the research, future studies could include a more diverse range of participants, such as high school students from eastern and western regions of China, or students from other educational stages. This expansion would help to identify any limitations that may exist when generalizing the research findings to a broader population, and it would contribute to a more comprehensive understanding of the current research issue.

Third, this study adopted a cross-sectional study design, which could not determine the causal relationship between the variables. In order to more accurately understand the relationship between teacher autonomy support, student feedback literacy, basic psychological needs and intrinsic motivation, future research can adopt longitudinal study design and conduct long-term follow-up research.

Finally, this study focuses on the general effect and takes little account of individual differences. For example, an individual’s personality characteristics may differ in the degree of need for autonomous support. Future research could further explore the responses and needs of students with different personality types for autonomous support to gain a more comprehensive understanding.

Despite the above limitations, this study has obtained useful insights and enlightenments by studying the multiple mediating roles of teachers’ autonomous support, basic psychological needs and intrinsic motivation in high school students’ feedback literacy. Future research can further expand our understanding of this field and take corresponding improvement measures to improve the reliability and wide applicability of the research.

## Conclusion

Based on previous empirical studies on teachers’ strategies to support feedback to improve students’ feedback literacy, this study explores the mechanism of the relationship between teachers’ support autonomy and high school students’ feedback literacy in feedback. The research results show that teachers’ autonomous support in feedback not only directly promotes the development of high school students’ feedback literacy, but also influences the feedback literacy of high school students through influencing basic psychological needs and intrinsic motivation, and through the chain relationship between them. In addition, this study supplemented the relationship among these influencing factors to some extent, enriched our understanding of the comprehensive impact of teachers’ autonomous support, basic psychological needs and intrinsic motivation on feedback literacy, and provided a new perspective for promoting the development of high school students’ feedback literacy and carrying out corresponding teaching interventions.

On this basis, this paper puts forward the following suggestions: First, teachers should consciously integrate autonomous support into teacher-student feedback and peer feedback, so as to promote the development of senior high school students’ feedback literacy in regular learning. Teachers should strive to create a self-supporting learning environment, provide a learning atmosphere where students can explore independently, and adopt self-supporting teaching methods and means to enhance the effect of feedback. Second, teachers should pay attention to the important role of basic psychological needs and intrinsic motivation in the relationship between teachers’ autonomous support and students’ feedback literacy. In particular, attention should be paid to the satisfaction of students’ basic needs and the stimulation of intrinsic motivation. Teachers should adopt a variety of methods and means to promote the satisfaction of students’ psychological needs, stimulate students’ learning motivation and interest, and cultivate internal motivation resources to promote effective feedback process. Third, teachers should conduct more in-depth practical exploration, enrich the strategies and methods of autonomous support feedback, and summarize the effective ways to meet the basic psychological needs and stimulate the internal motivation, so as to improve the timeliness of teacher-student feedback and peer feedback. Fourth, it is suggested to carry out practical training intervention of feedback literacy to improve high school students’ feedback perception ability, feedback judgment ability, emotion management ability and improve the action ability of deep learning. These suggestions are designed to help teachers better support and cultivate students’ feedback literacy in the feedback process, while providing practical and feasible teaching strategies and methods.

## Data availability statement

The raw data supporting the conclusions of this article will be made available by the authors, without undue reservation.

## Ethics statement

The studies involving humans were approved by Shaanxi Normal University ethics committee. The studies were conducted in accordance with the local legislation and institutional requirements. Written informed consent for participation in this study was provided by the participants’ legal guardians/next of kin.

## Author contributions

SZ: Writing – original draft. JX: Writing – review & editing. HC: Writing – review & editing. LJ: Writing – review & editing. XY: Writing – review & editing.
